# Space environment adaptability of 2D semiconductor materials

**DOI:** 10.1093/nsr/nwaf064

**Published:** 2025-02-21

**Authors:** Lingxiao Yu, Shiran Sun, Yi Jia, Feiyu Kang, Ruitao Lv

**Affiliations:** State Key Laborotary of New Ceramic Materials, School of Materials Science and Engineering, Tsinghua University, China; State Key Laborotary of New Ceramic Materials, School of Materials Science and Engineering, Tsinghua University, China; China Academy of Aerospace Science and Innovation, China; Shenzhen Geim Graphene Center, Institute of Materials Research, Tsinghua Shenzhen International Graduate School, Tsinghua University, China; Key Laboratory of Advanced Materials (MOE), School of Materials Science and Engineering, Tsinghua University, China; State Key Laborotary of New Ceramic Materials, School of Materials Science and Engineering, Tsinghua University, China; Key Laboratory of Advanced Materials (MOE), School of Materials Science and Engineering, Tsinghua University, China

## Abstract

Two-dimensional semiconductors demonstrate unprecedented stability in extreme space conditions, enabling next-generation electronics for advanced space technologies in harsh cosmic environments.

Space exploration plays a pivotal role in advancing human technological progress and deepening our understanding of the universe [1]. Satellites, as key instruments in space exploration, not only play an essential role in communication, navigation and meteorological monitoring, but also provide indispensable data for scientific research, environmental monitoring and national security. To ensure that these satellites operate reliably under the extreme conditions of space, one of their core electronic devices are transistors, which are widely used in the imaging systems and sensors of satellites [2]. The traditional silicon-based transistors are gradually approaching their theoretical limits due to the short-channel effect and strong carrier scattering caused by rough surfaces [3]. Alternatively, 2D transition metal dichalcogenides (TMDCs) have emerged as promising candidates for next-generation semiconductors due to their attractive properties, including atomic thickness, high charge-transfer efficiency, low power consumption, superior stability, large specific surface area, adjustable band gaps and significant exciton binding energy [4]. These advantages make TMDCs highly attractive for applications in electronics, optoelectronics and spintronics, offering vast potential for development and research [5]. To the best of our knowledge, there have been no reports on the space adaptability of 2D semiconductor materials and devices so far.

Given that satellites primarily operate in outer space, the 2D materials that are on them are also exposed to challenges such as radiation damage and temperature fluctuations. Optimizing these materials to withstand the extreme conditions of space remains a critical issue that researchers are urgently working to address. On 27 September 2024, China successfully launched its first reusable recoverable satellite, the Shijian-19. After 14 days in orbit, the satellite returned to Earth on 11 October 2024. 2D materials and field-effect transistors (FETs) have been sent into space by loading them onto the satellite. Our team intends to conduct in-depth research on the adaptability of 2D TMDCs in space environments and explore their potential applications in next-generation satellite technologies, which holds significant scientific and practical importance.

According to the latest developments in research, chemical vapor deposition (CVD) has become a key technique for synthesizing high-quality TMDCs due to its ability to precisely control material thickness, uniformity and crystal quality [6]. Herein, we first conducted research on the space environment adaptability of CVD-grown TMDC materials and the fabricated FETs that were based on WSe_2_ and Nb-doped WSe_2_, which were loaded into the Shijian-19 space capsule (between the aluminum skin and the thermal insulation layer) and directly exposed to the harsh environment of space, including radiation, microgravity and high/low temperatures, during the 14-day orbital flight. The polymer in the thermal insulation materials volatilized at high temperatures and was deposited onto the surface of materials and devices as the satellite passed through the atmosphere, leading to severe contamination. For the CVD-grown Nb-doped WSe_2_, to confirm that the Nb was indeed doped into WSe_2_, the corresponding X-ray photoelectron spectroscopy (XPS) and high-angle annular dark-field scanning transmission electron microscopy (HAADF-STEM) images are presented in Fig. S1. Upon the recovery of the satellite, the same testing positions were selected for the materials and devices for optical and electrical measurements to further evaluate the space environment adaptability of the samples. The schematic, photograph and optical microscope images of the devices are shown in Fig. 1a–g. Detailed growth parameters, device fabrication methods and optical microscope images of the devices (Fig. S2) are provided in the online Supporting Information. The schematic diagram of the back-gate FET is illustrated in Fig. 1a in which the back gate and the dielectric layer are composed of Si and 300-nm-thick SiO_2_, respectively. Additionally, a channel length of 4 μm was defined for the device, with 50-nm-thick Au electrodes serving as the source and drain electrodes transferred onto monolayer WSe_2_ (Fig. 1b) and Nb-doped WSe_2_ (Fig. 1c). In comparison with Fig. 1d and e, the severe contamination on the device surface after satellite recovery (Fig. 1f and g) indicates the harsh conditions of the testing environment.

**Figure 1.**
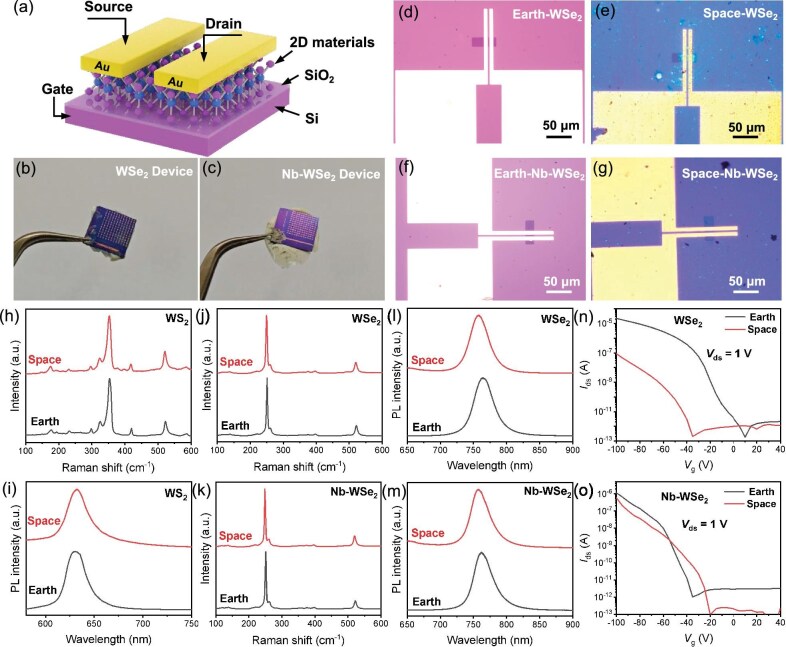
Comparisons of 2D semiconductor materials and devices before and after in-orbit flight test in space. (a) Schematic diagram of the as-fabricated back-gate FET based on 2D materials. (b and c) Photographs of (b) WSe_2_ and (c) Nb-doped WSe_2_ back-gate FETs after space test. (d–g) Optical microscope images of (d and e) WSe_2_ and Nb-doped (f and g) WSe_2_ FETs before and after space test. (h) PL spectra and (i) Raman spectra of CVD-grown WS_2_ flakes before and after space test. (j and k) Raman spectra of (j) CVD-grown WSe_2_ and (k) Nb-doped WSe_2_ flakes before and after space test. PL spectra of (l) CVD-grown WSe_2_ and (m) Nb-doped WSe_2_ flakes before and after space test. (n and o) Transfer characteristics (*I*_ds_−*V*_g_) swept gate voltages from −100 to 40 V of the FETs based on (n) monolayer WSe_2_ and (o) Nb-doped WSe_2_ before and after the space test.

Optical characterization, including photoluminescence (PL) and Raman spectroscopy, was performed to demonstrate the influence of the space test on 2D semiconductors such as WS_2_, WSe_2_ and Nb-doped WSe_2_ before and after the in-orbit flight test. In all spectra, the red curves represent data from samples that have been exposed to the space environment while the black curves correspond to samples before space loading. No significant differences in the optical properties of our samples are observed before and after the flight test. The Raman spectrum of CVD-grown WS_2_ typically shows a sharp E^1^_2__g_ peak at 356 cm^−1^ and an A_1__g_ peak at 417 cm^−1^, along with several secondary and multimodal overlapping peaks (Fig. 1h), while the PL peaks are both at ∼630 nm and the full-width half-maximums (FWHMs) are both 24 nm (Fig. 1i) for the ‘Earth’ and ‘space’ graph lines. When it comes to WSe_2_, the Raman spectrum shows a sharp E^1^_2__g_ peak at ∼250.3 cm^−1^ and an A^1^_g_ peak at ∼260.3 cm^−1^ (Fig. 1j). Both the Raman peak positions of WS_2_ and WSe_2_ align with the standard data reported in the literature [7,8]. The PL spectra for CVD-grown WSe_2_ are shown in Fig. 1l. The monolayer WSe_2_ exhibits strong emission at ∼760 nm and the FWHMs are both ∼34 nm for the two spectra. In the case of Nb-doped WSe_2_, while the number of Raman characteristic peaks and vibrational modes remain unchanged before and after the space flight test (Fig. 1k), the FWHMs also show minimal differences in PL spectra (Fig. 1m). Thus, all optical characterizations confirm that the optical and crystal qualities of CVD-grown materials are well preserved in the space environment, demonstrating terrific stability.

The electrical performance of FETs was also measured to elaborate the space adaptability of 2D transistors. Back-gate FETs were fabricated based on CVD-grown monolayer WSe_2_ and Nb-doped WSe_2_, respectively, via the electrode-transfer method. The *I*_ds_–*V*_g_ transfer characteristics were measured before and after the satellite launch. Figure 1n and o shows the *I*_ds_–*V*_g_ transfer characteristics in the range of 40–100 V. All curves exhibit typical p-type semiconductor behaviors. Although the on-current decreased after the harsh environment test, the devices still maintained good semiconductor-switching characteristics. For the WSe_2_-based device (Fig. 1n), the on-current decreased by approximately one order of magnitude and the off-current remained at <10^−12^ A/μm before and after exposure to space. The decrease in the on-current is attributed to prolonged exposure to the air and severe surface pollution (as shown in Fig. 1e and g) during the loading process; thus the device exhibited a decrease in the on-/off-current ratio after the satellite flight, yet it was still up to a level of ∼10^6^, as shown in Fig. 1n. For the Nb-doped WSe_2_-based device (Fig. 1o), the on-/off-current ratio of the device shows little change before and after space environment exposure. The impact of space testing on the SiO_2_ dielectric layers was also investigated; the leakage current of the dielectric layer (SiO_2_) was in the picoampere (pA) range before the space flight test and increased to the nanoampere (nA) range after the test (Fig. S5), indicating that its insulating properties had deteriorated due to the radiation-induced defects in the SiO_2_. Overall, the electrical properties of the 2D semiconductor devices show little variation even after undergoing the harsh space environment during orbital flight. The CVD-grown materials and FET devices demonstrate superior adaptability to space conditions, holding great promise for future space applications.

In addition to the harsh space environment tests, supplementary tests were also conducted in the spacecraft interior to demonstrate the further potentials of 2D semiconductor materials. Here, we utilized CVD-grown WSe_2_ and MoSe_2_ with different layers as the examples. The samples from the same growth batch were divided into two groups: one was placed inside the space capsule and the other was stored under ambient conditions on Earth. After the satellite returned, Raman and PL characterization were performed on both sets of samples to eliminate the effects of growth and time, serving as comparative controls. As shown in Figs S3 and S4, the red curves represent the samples that were stored in the space capsule and the black curves represent the samples that were stored on Earth. The Raman spectra of the Earth and space samples show minimal differences, while the PL intensity of the space-exposed samples is higher than that of the Earth-stored samples and the FWHMs also have little change. The relatively low PL intensities of the samples on Earth may be attributed to the effects of water and oxygen under an ambient environment. Overall, the optical properties of both WSe_2_ and MoSe_2_ demonstrated the high adaptability of 2D semiconductors under space conditions.

In summary, we demonstrate the superior adaptability of 2D TMDC materials on both optical and electrical performance under the complex conditions of the space environment. The 2D transistors also maintained good semiconductor-switching characteristics after the space test. Our findings open up new possibilities for their application in advanced space technologies, such as efficient photovoltaics, radiation-resistant electronic devices and high-sensitivity optical sensors, and deepen our understanding of material–environment interactions, which are crucial for designing more 2D materials that are suited to space conditions. Therefore, the adaptability of 2D materials to the space environment not only highlights their potentials in next-generation aerospace applications, but also inspires further exploration of their use in other extreme environments on Earth. This research represents a fundamental step in bridging material science with future space exploration and technological needs.

## Supplementary Material

nwaf064_Supplemental_File
