# Supplementary material for: Space environment adaptability of 2D semiconductor materials
Source: Natl Sci Rev. 2025 Feb 21;12(4):nwaf064. doi: 10.1093/nsr/nwaf064 (PMC11932342; doi:10.1093/nsr/nwaf064)
Supplement: nwaf064_Supplemental_File [file nwaf064_supplemental_file.pdf]

Support Information

Space Environment Adaptability of Two-dimensional Semiconductor Materials

Lingxiao Yu[1]†, Shiran Sun[1]†, Yi Jia[2]†, Feiyu Kang[3,4] and Ruitao Lv[1,4]*

[1]State Key Laborotary of New Ceramic Materials, School of Materials Science and Engineering, Tsinghua University, Beijing 100084, China

[2]China Academy of Aerospace Science and Innovation, Beijing 100088, China

[3]Shenzhen Geim Graphene Center, Institute of Materials Research, Tsinghua Shenzhen International Graduate School, Tsinghua University, Shenzhen 518055, China.

[4]Key Laboratory of Advanced Materials (MOE), School of Materials Science and Engineering, Tsinghua University, Beijing 100084, China.

*Corresponding Author:

Ruitao Lv Email: lvruitao@tsinghua.edu.cn

†These authors contributed equally to this work.

Some Background for the Project of Reusable Recoverable Satellite

On October 10, 2024, at 10:39 PM, China's first reusable recoverable satellite, the Shijian-19, successfully landed at the Dongfeng Landing Site after completing a two-week low Earth orbit (LEO) mission. Specifically, the satellite carried nine space science experimental payloads, including research on two-dimensional semiconductor materials and devices, oral medical materials and the molecular mechanisms of microbial drug production, etc. The Shijian-19 Satellite provided an opportunity for conducting space experiments and enabled in-orbit testing and validation of domestically developed components and raw materials, thereby promoting the development and application of new space technologies. This mission significantly

advanced research in fields such as semiconductor materials, microgravity science and space life sciences. Moreover, the successful recovery of the satellite marked a major breakthrough in reusable recoverable satellite technology, laying a solid foundation for future missions in China's space exploration endeavors.

Synthesis of WSe_2 and Nb-doped WSe_2

The few-layer WSe_2 and Nb-doped WSe_2 films were prepared via atmospheric pressure chemical vapor deposition (APCVD). For the synthesis, 40 mg of Na_2WO_4 (or a mixture of 40 mg Na_2WO_4 and 4 mg $C_{10}H_5NbO_{20}$ for Nb-doped WSe_2) was dissolved in 10 mL of a 1:1 (v/v) mixture of ethanol and deionized water. A 10 μL aliquot of this solution was spin-coated onto a SiO_2/Si substrate (2 cm×2 cm) pre-treated with O_2 plasma (100 W, 60 s) at 3000 rpm for 1 minute. The coated substrate was dried and placed on a quartz boat at the center of a reaction tube. Separately, 300 mg of Se powder was evenly distributed on another quartz boat upstream, covering an area ranging from 1 cm^2 to 4 cm^2.

After purging the system with 900 sccm of Ar for 10 minutes, the temperature was ramped to 800 ℃ over 30 minutes under a flow of 180 sccm Ar. During this process, all precursors were kept outside the heating zone. Once the furnace reached 800 ℃, the substrate-containing quartz boat was moved into the heating zone. Following a pre-annealing period of 5 minutes, the quartz boat containing the Se powder was introduced into the furnace. When the Se powder began to melt, a gas mixture of 180 sccm Ar and 6 sccm H_2 was introduced and maintained for 3 minutes. Finally, the quartz boat was rapidly withdrawn from the furnace and cooled down to room temperature under a continuous flow of 100 sccm Ar.

Synthesis of WS_2

First, 1.5 mg/mL NaCl solution was prepared, and 2 μL of the solution was dropped onto a O_2-plasma treated SiO_2/Si substrate. After drying, 3 mg of WO_3

powder was placed on NaCl. A second O_2-plasma-treated SiO_2/Si substrate was then placed approximately 3 mm above it on a quartz boat and placed in the center of the tube. A second quartz boat, containing 900 mg of sulfur powder, was positioned ~30 cm upstream. The tube furnace, used for the initial growth step, had a 2-inch diameter and a 2-meter length, and was a single-zone furnace. During the synthesis, the argon flow rate was set to 100 sccm, the heating rate was 20 ℃/min, and the growth temperature was 780 ℃, with a 10-minute growth time. Due to the high melting point of the tungsten source, a longer evaporation time for sulfur was required to achieve uniform evaporation. As a result, the tungsten precursor was preheated with the furnace to the target temperature before introducing the sulfur powder for WS_2 growth. After the reaction, the furnace was quickly cooled to room temperature under 100 sccm Ar.

Synthesis of $MoSe_2$

$MoSe_2$ was synthesized via CVD. In a typical process, 30 mg of MoO_3 and 15 mg or 30 mg of KI were dissolved in 10 mL of deionized water to grow monolayer and bilayer $MoSe_2$, respectively. The mixture was sonicated for 30 minutes to form a suspension. A 10 μL aliquot of this suspension was deposited onto a pre-treated SiO_2/Si substrate. A second O_2-plasma-treated SiO_2/Si substrate was then placed approximately 2 mm (1 mm for bilayer $MoSe_2$) above it. 600 mg of Se powder was positioned upstream. The subsequent growth procedure followed the same steps as for WSe_2.

Device Fabrication via vdW Metal-Semiconductor Contact Integration

To address the issue of large gate current leakage caused by pinholes and microcracks in the dielectric layer after high-temperature CVD growth, the synthesized samples were transferred onto a fresh SiO_2 (300 nm)/Si substrate. Field-effect transistors (FETs) with van der Waals (vdW) integrated contacts were fabricated using an electrode transfer technique. The electrodes were patterned via laser direct writing lithography (LDWL), followed by the deposition of 50 nm of Au onto the SiO_2 /Si substrate through electron beam evaporation. After completing the photoresist lift-off process, the electrodes were cleaned using ultrasonic treatment in

deionized water for 60 seconds to remove solvent residues and weaken the adhesion between the Au electrodes and the SiO_2/Si substrate. Subsequently, O_2 plasma treatment was applied to promote water molecule intercalation.

The transfer process for Au electrodes was analogous to that used for 2D materials. The pre-treated Au electrodes were spin-coated with an 8 wt% polycarbonate (PC) chloroform solution at 3000 rpm for 1 minute, followed by baking at 80 ℃. After cooling down to room temperature, the edges of the substrate were scraped to expose the PC film, and thermal release tape (TRT) was adhered to the substrate. Deionized water was then applied along the scraped edges to facilitate the detachment of the TRT/PC/electrode assembly from the substrate. The detached assembly was dried using an N_2 flow to remove water droplets from the PC surface, then adhered to a polydimethylsiloxane (PDMS) stamp. Using a micromechanical aligner, the TRT/PC/electrode assembly was transferred to the target substrate. The TRT and PDMS stamp were released at 120 ℃, and the assembly was further heated up to 150 ℃ to eliminate residual bubbles and ensure robust contact between the electrodes and the sample. Finally, the PC layer was dissolved in chloroform after cooling to room temperature.

Optical Measurement

Optical characterizations were conducted using Raman and photoluminescence spectroscopy with the LabRAM HR Evolution confocal Raman spectrometer (Horiba) on SiO_2/Si substrates. A 532 nm laser focused through a 50× objective lens was used as the excitation source.

Electrical Measurement

The electrical performance test required a semiconductor parameter analyzer (Keysight B1500A) and a probe station was equipped with optical microscopes under vacuum. The probe station established a conductive channel between the device and

the semiconductor analyzer, which applied DC voltage signals to the three channels and collected the current signals.

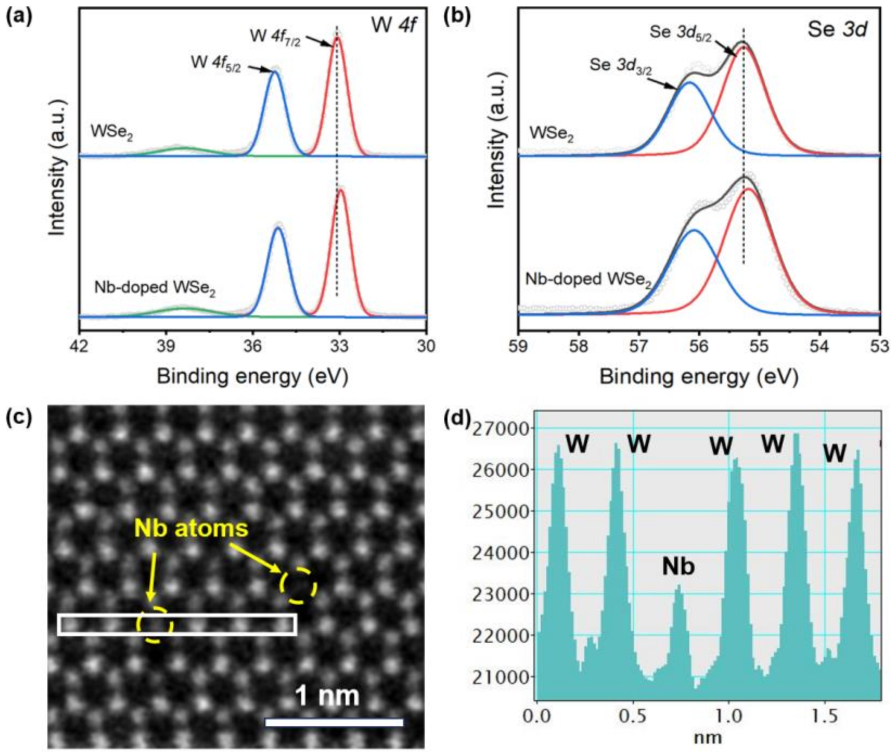

Figure S1 Materials characterizations of WSe_2 and Nb-doped WSe_2. (a) W 4f XPS fine scan spectra of WSe_2 and Nb-doped WSe_2. (b) Se 3d XPS fine scan spectra of WSe_2 and Nb-doped WSe_2. (c) HAADF-STEM images of Nb-doped WSe_2. (d) intensity profile of white box in (c). For Fig. S1(a) and (b), compared to WSe_2, both W $4f_{7/2}$ peak and Se $3d_{5/2}$ peak in Nb-doped WSe_2 shift towards lower binding energy. The peak shifts are attributed to the doping of Nb. From Fig. S1(c), it can be observed that Nb enters WSe_2 in the form of substitutional doping. Additionally, since elements with higher atomic numbers display higher brightness in the image, combined with Fig. S1(d), atoms in the darker position correspond to Nb with a lower atomic number.

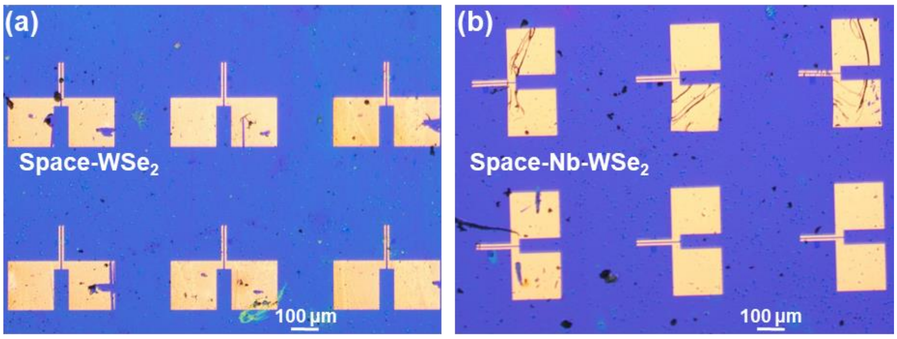

Figure S2 Optical microscope images of back-gate vdW integrated FET device (taken after the satellite's recovery) (a) WSe_2-based FET device (b) Nb-doped WSe_2-based FET device.

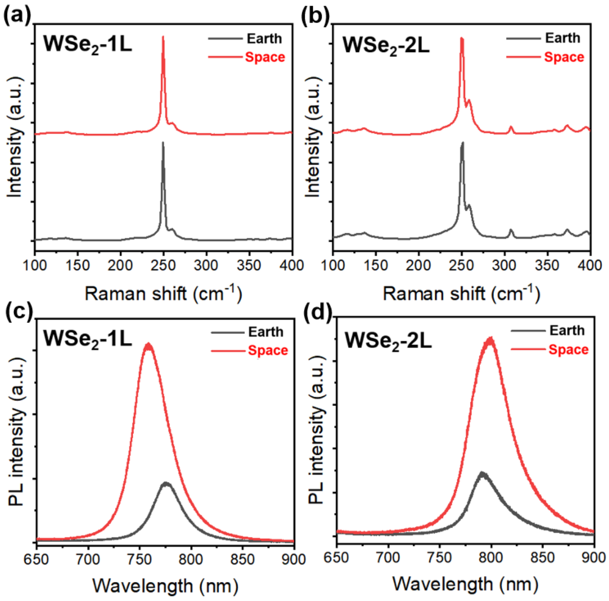

Figure S3 Raman and PL spectra of monolayer and bilayer WSe_2 (Earth : Captured and tested before the satellite launched; Space: Captured and tested after the satellite recovered) (a) Raman spectra of monolayer WSe_2 (b) Raman spectra of bilayer WSe_2 (c) PL spectra of monolayer WSe_2 (d) PL spectra of bilayer WSe_2.

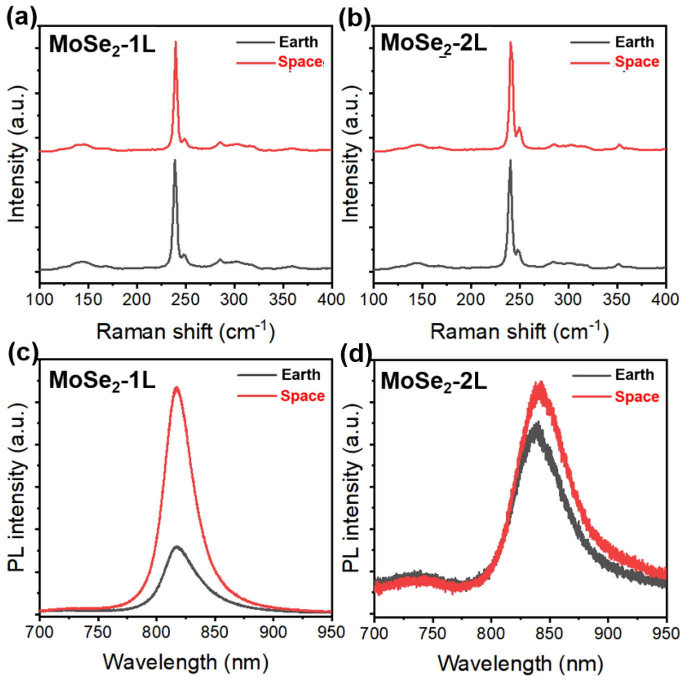

Figure S4 Raman and PL spectra of monolayer and bilayer $MoSe_2$. (Earth: Captured and tested before the satellite launched; Space: Captured and tested after the satellite recovered) (a) Raman spectra of monolayer $MoSe_2$. (b) Raman spectra of bilayer $MoSe_2$. (c) PL spectra of monolayer $MoSe_2$. (d) PL spectra of bilayer $MoSe_2$.

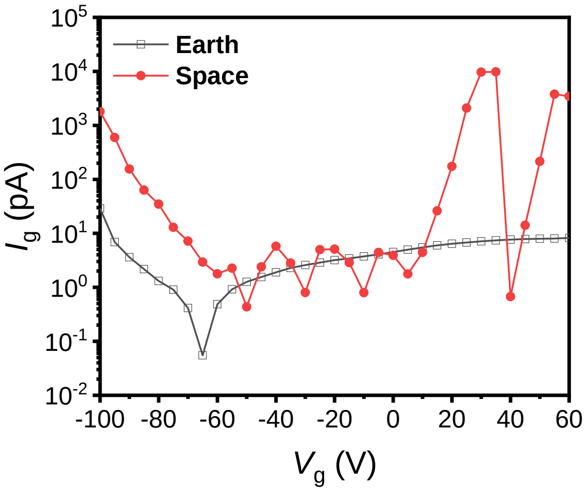

Figure S5 Comparison of leakage current of the SiO_2 dielectric layer before and after the space flight test. The leakage current has increased by two orders of magnitude after space flight, indicating the deterioration of insulating properties.
